# Effect of the Intake of Isoflavones on Risk Factors of Breast Cancer—A Systematic Review of Randomized Controlled Intervention Studies

**DOI:** 10.3390/nu13072309

**Published:** 2021-07-05

**Authors:** Luisa Finkeldey, Elena Schmitz, Sabine Ellinger

**Affiliations:** 1 Faculty of Nutrition and Food Sciences, Niederrhein, University of Applied Sciences, Rheydter Street 277, 41065 Mönchengladbach, Germany; luisa_finkeldey@web.de (L.F.); Schmitz-Elena7@gmx.de (E.S.); 2 Department of Nutrition and Food Sciences, Human Nutrition, University of Bonn, Meckenheimer Allee 166a, 53115 Bonn, Germany

**Keywords:** soy, isoflavone intake, biomarkers related to breast cancer, breast density, proliferation, inflammation, prevention, randomized controlled trials

## Abstract

Epidemiological studies suggest that high intake of soy isoflavones may protect against breast cancer, but causal relationships can only be established by experimental trials. Thus, we aimed to provide a systematic review of randomized controlled trials (RCTs) on the effect of an isoflavone intake on risk factors of breast cancer in healthy subjects. After a systematic literature search in PubMed, 18 different RCTs with pre- and/or postmenopausal women were included and investigated for details according to the PRISMA guideline. In these studies, isoflavones were provided by soy food or supplements in amounts between 36.5–235 mg/d for a period of 1–36 months. Breast density, estrogens including precursors, metabolites, estrogen response such as length of menstrual cycle, and markers of proliferation and inflammation were considered. However, in most studies, differences were not detectable between isoflavone and control/placebo treatment despite a good adherence to isoflavone treatment, irrespective of the kind of intervention, the dose of isoflavones used, and the duration of isoflavone treatment. However, the lack of significant changes in most studies does not prove the lack of effects as a sample size calculation was often missing. Taking into account the risk of bias and methodological limitations, there is little evidence that isoflavone treatment modulates risk factors of breast cancer in pre- and postmenopausal women. Future studies should calculate the sample size to detect possible effects and consider methodological details to improve the study quality.

## 1. Introduction

Breast cancer is the most frequent type of cancer in women globally. Each year, about 2.3 million women worldwide develop breast cancer, and about 685,000 people were estimated to die from breast cancer in 2020 [1]. Women from Europe and North America are particularly concerned as the age-adjusted incidence rate of breast cancer is approximately 2–4 times higher than in Asia [2].

Breast cancer is favored by race, ethnicity, family history of cancer, genetic variants and mutations of genes modulating DNA repair. Age at menarche, parity, as well as age at first pregnancy affect the risk by modulating the long-term sex hormone levels. Physical inactivity is a modifiable risk factor as well as diet. Whereas certain foods and food ingredients increase the risk of breast cancer (e.g., alcohol), others like soy or isoflavones seems to be protective [3].

More than 25 years ago, a population-based case-control study revealed a higher breast cancer rate in women of Chinese, Japanese, and Filipino ethnicities migrating to USA and Hawaii than in the countries of origin, which approximated in Asian-Americans born in the West to the U.S. white rate. However, the risk increased among Asian immigrants in the U.S. over several generations, suggesting that the Western lifestyle substantially increased the breast cancer risk [4].

In contrast to the Western diet, the Asian diet is traditionally rich in foods produced from soybeans as main ingredients. As soy products are the major dietary source of isoflavones, the intake of isoflavones in Asia (China: 6.2–75.7 mg/d; Japan: 22.6–54.3 mg/d) is much higher than in Europe (0.37–4.5 mg/d) and in USA (0.73–3.3 mg/d) despite considerable variation between individual studies [5]. A meta-analysis mainly derived from case-control studies has shown that a high (≥20 mg/d) and moderate isoflavone intake (~10 mg/d) by consumption of soy food reduces the risk of breast cancer in Asia and Asian American populations by 29% and 12%, respectively, compared to a low isoflavone intake (≤5 mg/d). This effect was dose-dependent (risk reduction about 16% per 10 mg of isoflavones intake per day). Moreover, it could be observed in both pre- and postmenopausal women. However, in Western populations, high vs. low isoflavone intake (≥0.8 mg/d vs. ≤0.15 mg/d) did not affect the breast cancer risk [6]. In contrast, a recently published meta-analysis of 16 prospective cohort studies (six from Asia, 10 from Western countries) did not find an association between high and moderate vs. low isoflavone intake and the risk of breast cancer. However, if consumption of soy food was considered, high vs. low intake of soy foods was associated with a 13% lower risk to develop breast cancer. In addition, moderate consumption of soy food was also associated with a 25%–28% reduced breast cancer risk if the duration of the follow-up lasted ≥10 years, and if the study was adjusted for smoking status, alcohol intake, and for hormone replacement therapy [7].

Isoflavones such as genistein and daidzein may protect against breast cancer through certain mechanisms. First, isoflavones may affect the hormone levels in breast and ovaries by modulating the activity of steroidogenic enzymes (e.g., aromatase, 3- and 17β-hydroxysteroid dehydrogenase), thereby reducing the conversion of estrogen precursors (androgens) to estrogens and the dehydrogenation of estrone (E_1_) to estradiol (E_2_). Second, isoflavones may alter estrogen metabolism away from cancerous metabolites (16-α-hydroxyl metabolites) towards 2-hydroxy estrogen metabolites with lower estrogen activity. Third, due to structural similarities to human 17β-estradiol, isoflavones can bind to estrogen receptors (ER), preferentially to ER-β, which suppresses the transcription of many genes involved in cell growth and inflammation, thereby diminishing the estrogenic effects induced by ERα [8,9].

As reviewed earlier, isoflavones may also prevent against breast cancer by other mechanisms. Binding of phytoestrogens to ER at the surface of cells might directly modulate the expression of genes by the inhibition of signaling pathways like Akt and MAPK stimulating cell growth, proliferation, and survival, while activating proapoptotic genes like Bcl-2, p53, caspase-3, Bax, BRCA-1, and BRCA-2 [10]. By this mechanism, isoflavones may stimulate the synthesis of sex hormone binding globulin (SHBG), thereby reducing the free (active) E_2_ in plasma [11].

Moreover, isoflavones exert antioxidant properties [12,13]. Since reactive oxygen species from exogenous and endogenous sources (estrogens) favor oxidative stress which in turn can stimulate inflammatory and proliferative pathways involved in the pathogenesis of breast cancer [14], the antioxidant and anti-inflammatory properties of isoflavones might also contribute to protection from breast cancer.

Hence, epidemiological studies suggest that high isoflavone intake may protect against breast cancer. This is supported by in vitro studies on biological mechanisms to explain how isoflavones can modulate steps involved in the development of breast cancer. However, the effectiveness of isoflavone treatment for the prevention of breast cancer can only be investigated by intervention studies with a randomized controlled design which allow cause–effect relationships between intervention and outcome [15]. Randomization balances patients’ characteristics between the groups or treatments and enables attribution of any differences in outcome to intervention [16]. As breast cancer develops over years, the response to isoflavone intervention can only be evaluated by surrogate endpoint markers, i.e., by risk factors for breast cancer that can easily be determined by non-invasive methods [17].

Breast density is an independent risk factor for breast cancer [18]. Risk factors in serum include free E_2_, insulin-like growth factor 1 (IGF-1), the ratio of IGF-1 to IGF binding protein 3 (IGFBP-3), single nucleotide polymorphisms, and breast intra-epithelial neoplasia. An increased breast density is associated with changes in the microenvironment (increased secretion of inflammatory molecules, cytokines, growth factors) that favor tumor growth [19], and with a reduced renal excretion of estrogens [20]. Estrogens stimulate the expression of genes involved in cell growth and inflammation [21]. Ki-67 antigen, expressed by proliferating cells, is an established molecular marker for breast cancer [22]. Nipple aspiration fluid (NAF) cytology is considered as predictor for breast cancer [23], with a high diagnostic specificity (0.97), but a low sensitivity (0.64) which limits diagnostic accuracy [24]. As hormonal stimulation of the breast tissue plays a considerable role in breast carcinogenesis and for the length of the menstrual cycle (MC), the latter might be relevant for breast cancer risk [25].

Hence, the aim of this systematic review was to investigate whether the intake of isoflavones by healthy subjects in RCTs may protect against breast cancer by consideration of breast density (main endpoint). In addition, further parameters associated with the risk of breast cancer (estrogens, growth factors, markers on inflammation, proliferation and apoptosis, NAF cytology, length of MC) were assessed. Moreover, the risk of bias (RoB) and the imprecision of the included studies was judged to evaluate the evidence for preventive effects.

## 2. Materials and Methods

This systematic review was conducted according to the Preferred Reporting Items for Systematic Review and Meta-Analysis (PRISMA) guidelines [26].

### 2.1. Literature Search Strategy

A systematic literature search was performed in PubMed for human intervention studies that investigated the effects of isoflavone intake on risk factors of breast cancer. For the literature search, the following combinations of keywords were used: “breast cancer soy”, “breast cancer isoflavones prevention”, “breast cancer phytoestrogen prevention”, “breast cancer red clover”, “mammary carcinoma soy”, “mammary carcinoma isoflavones prevention”, “mammary carcinoma phytoestrogen prevention”, “mammary carcinoma red clover”, “breast cancer isoflavones primary prevention”, “breast cancer phytoestrogen primary prevention”, “breast cancer phytoestrogen primary prevent *”. Filters were applied with regard to study type (RCTs), language (German, English), and species (humans). The database search was performed by two reviewers (L.F., E.S.) up to 31 July 2020 for relevant studies that were published as original contributions or short communications. Additional databases beyond PubMed were not used for literature search as for other clinical topics, the search in PubMed has shown a higher specificity than Google Scholar, and a comparable sensitivity, suggesting that PubMed is an optimal tool for biomedical research [27]. Paid databases such as Scopus and Web of Science cover further areas of research (e.g., health, life and social sciences, technology) and have shown to provide additional records, but mostly without relevance for biomedical questions [28].

### 2.2. Inclusion and Exclusion Criteria

Studies were included if (1) they investigated the effect of an isoflavone intake on risk factors of breast cancer, e.g., breast density, estrogen metabolites, length of MC, markers of proliferation, volume and cytology of NAF, tyrosine kinase activity and expression of genes related to proliferation, apoptosis, and estrogenic effects. Further inclusion criteria were (2) a randomized controlled study design, (3) isoflavone treatment by consumption of soy foods or supplements, (4) a control treatment with restrictions on soy consumption or isoflavone intake or an isoflavone-free placebo if isoflavones were provided as supplements (e.g., capsules, tablets, soy protein powder). Exclusion criteria comprised (1) the investigation of subjects already suffering from breast cancer, (2) treatment of the subjects with oral contraceptives, and (3) in placebo-controlled studies, the use of a low-isoflavone intake instead of an isoflavone-free placebo treatment.

### 2.3. Study Selection, Data Extraction, and Assessment of Study Quality

Two independent reviewers (L.F., E.S.) identified relevant studies according to the predefined eligibility criteria. All records were checked for duplicates. Duplicates were removed and the remaining records were screened by title and/or abstract to exclude records that did not meet the inclusion criteria. For the remaining records, the full-text articles of potentially relevant studies were checked for eligibility, based on the above-mentioned criteria. Any discrepancies in the study selection process were discussed between both reviewers (L.F., E.S.) and if necessary with S.E., until a consensus was reached. Finally, eligible trials were included in this review.

Both review authors (L.F., E.S.) extracted relevant data from the included studies independently by using a self-made Excel template: study design, details on intervention (kind/amount of isoflavones, application form) and on control or placebo treatment, participants (sample size, demographic data, criteria of eligibility), country in which the study was performed, and risk factors of breast cancer. Moreover, parameters on the bioavailability of isoflavones and on the compliance with intervention (e.g., urinary isoflavone excretion) were considered. Moreover, studies were checked for sample size calculation (prospectively performed, biomarker chosen, sufficient subjects available for statistical evaluation) and checked for industry funding. Discrepancies in data extraction were discussed between both reviewers and, if necessary, with S.E.

Afterwards, the RoB of included RCTs was independently assessed by two authors (L.F., S.E.) using the Cochrane risk of bias tool [29] considering the following criteria: (1) generation of randomization list before the study and using an adequate randomization method (selection bias), (2) allocation concealment from participants and investigators, (3) blinding of participants and investigators (performance bias), (4) blinding of outcome assessment until completion of statistical evaluation (detection bias), (5) completeness of outcome data, reporting number and reasons of dropout for each group/treatment, application of intention-to-treat analysis or statistical models to consider missing values (attrition bias), (6) registration of the study protocol, reporting full endpoints and outcomes according to registration (reporting bias), (7) considering potential confounder from diet (other risk of bias) by investigation of food consumption, restrictions on soy or isoflavone intake, and by considering the compliance with intervention. The latter was investigated by urinary isoflavone excretion, by a diary where the intake of foods or supplements were documented, or as ratio of ingested capsules to the number of capsules that should have been ingested during intervention. The overall RoB was assessed within and across the studies by using the Cochrane risk of bias tool [29]. Each publication was checked for the registration number of the study protocol and each study for registration at clinicaltrials.gov. Again, a self-made Excel template was used to check these criteria for each study. Discrepancies in RoB assessment occurred if details on randomization, blinding of investigators and allocation concealment remained unclear. A closer look on the study design and/or on the results could be helpful. If the procedure to consider missing values was not clearly described, the statistical analysis was illuminated in detail. Discrepancies were also resolved through discussion.

## 3. Results

### 3.1. Study Selection and Study Characteristics

After a systematic literature search in PubMed, 162 records were identified. After removing duplicates, 78 records remained and were screened by title and/or by abstract. In total, 36 records were excluded after screening as being not relevant for the question addressed by this review. The remaining 42 records were assessed for eligibility by the full text. Thirteen intervention studies were excluded as they were not randomized (*n* = 2), had no adequate control/placebo treatment (*n* = 3) or did not address the question of the review (*n* = 8). Finally, 29 records were considered to be eligible [30,31,32,33,34,35,36,37,38,39,40,41,42,43,44,45,46,47,48,49,50,51,52,53,54,55,56,57,58].

**Figure 1:**
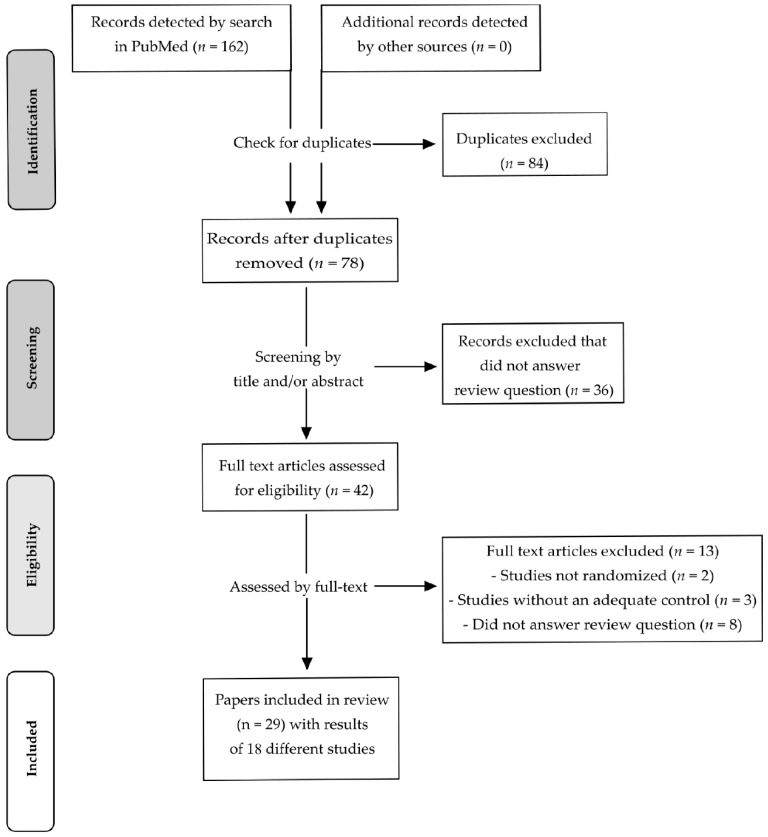
Flow-diagram of study selection process according to PRISMA statement.

However, with regard to country, participants, study design, intervention and study protocol registration, it becomes obvious that the results of Maskarinec et al., 2002a [34], Maskarinec et al., 2002b [35] and Maskarinec et al., 2003 [37] derived from a single study (study A). This also applies to Maskarinec et al., 2004a [36], Maskarinec et al., 2004b [38], Maskarinec et al., 2005 [40], and Maskarinec et al., 2009b [41]. These results are described in different publications and derived from a second study (study B). Maskarinec et al., 2011a [42], Maskarinec et al., 2011b [43], Morimoto et al., 2012 [45], Sen et al., 2012 [46], and Maskarinec et al., 2013 [47] present the results of a third study (study C) of the same working group. Maskarinec et al., 2012 [44] and Maskarinec et al., 2017 [48] also provided results which were partly obtained from study B and study C, respectively. Since 14 publications of Maskarinec and co-workers [34,35,36,37,38,40,41,42,43,44,45,46,47,48] were obtained from three different trials, the 29 records included in the present review described the results of 18 different RCTs. A flow diagram of the identification and selection of the studies is shown in Figure 1.

These RCTs were conducted in the USA [30,31,33,34,35,36,37,38,40,41,42,43,44,45,46,47,48,50,51,52,54,55,56,57], the Netherlands [49], Germany [39], the United Kingdom [55,56,58], Brazil [53], and Japan [32]. Participants were women in premenopausal [30,31,32,33,34,36,37,38,39,40,41,42,43,44,45,46,47,48] or postmenopausal state [49,50,51,52,53,54]. A few RCTs investigated a mixed group of pre-, post-, as well as perimenopausal women [55,56,57,58]. Isoflavones were provided by supplements [30,31,33,34,35,37,49,50,51,52,53,55,56,57,58] or soy food [32,36,38,39,40,41,42,43,44,45,46,47,48,54] in amounts between 36.5 and 235 mg/d. The isoflavone intake by supplements was about 60% higher than by soy food (85 ± 12 mg/d vs. 54 ± 5 mg/d; means ± SEM). The duration of intervention ranged between 1–36 months [30,31,32,33,34,35,36,37,38,39,40,41,42,43,44,45,46,47,48,49,50,51,52,53,54,55,56,57,58] and was comparable between studies providing isoflavones by supplements or soy foods (12 ± 2 vs. 11 ± 3 months; means ± SEM).

### 3.2. Studies with Premenopausal Women

Eighteen RCTs were conducted with premenopausal women. Details are shown in Table 1.

Nagata et al. [32] investigated the effect of soymilk consumption (400 mL/d; 109 mg/d isoflavones) for 3 MC in addition to a regular diet. The study was done in parallel group design in Japan. Soymilk consumption decreased E_1_ in serum after 3 MC compared to baseline and compared to control treatment. The changes in E_2_, SHBG and in the length of MC were not different between both treatments.

Zittermann et al. [39] provided soy-containing cookies (52 mg isoflavones/d) and soy-free cookies (placebo), respectively, daily for 1 MC in a crossover study. The urinary excretion of genistein and daidzein increased, but this was not accompanied by different concentrations of E_1_, E_2_ (total, free), follicle-stimulating hormone (FSH), SHBG, and progesterone in serum between both treatments.

Duncan et al. [31] investigated whether supplementation of isoflavones in doses of 2.0 mg/kg BW/d and 1.0 mg/kg BW/d by soy protein induces hormonal changes compared to a protein powder providing only traces of isoflavones (0.15 mg/kg BW/d; control). In this crossover study, each intervention was done for 3 MC plus 9 days, with a 3-week-washout between two interventions. In the midfollicular phase, E_1_ was lower after an isoflavone intake of 2.0 mg/kg BW/d vs. 1.0 mg/kg BW/d. No differences could be observed in E_1_ in early follicular, periovulatory, and midluteal phase. In the periovulatory phase, luteinizing hormone (LH) and FSH were lower after medium isoflavone intake vs. control. In other phases, no differences could be observed. Estrone sulfate (E_1_S) and progesterone were not modulated by any treatment in any phase of the MC. Dehydroepiandrosterone sulfate (DHEAS) reached higher concentrations in plasma after high vs. medium isoflavone intake, but testosterone, androstenedione, dehydroepiandrosterone (DHEA), SHBG, and prolactin and the length of MC, follicular and luteal phase in MC 2 and 3 were comparable between all interventions.

Brown et al. [30] provided 40 mg/d isoflavones with soy protein in addition to a Western diet and used a Western diet free from soy protein as control. In this crossover study, each intervention was done for 2 MC. Changes in serum E_1_, E_2_, E_1_S, progesterone, testosterone, androstenedione, DHEA, DHEAS, SHBG, prolactin (mid-follicular and mid-luteal phase), FSH (mid-follicular phase), and LH (mid-luteal phase) were not detectable. The excretion of hormonal metabolites such as 2-(OH)E_1_ and 16α-(OH)E_1_ in 48-h-urine and the 2-(OH)E_1_-to-16α-(OH)E_1_-ratio was also comparable and the length of MC not different between all treatments.

Kumar et al. [33] provided 40 mg/d isoflavones by soy protein or an isoflavone-free milk protein (placebo) for 3 MC. Changes were not different between both treatments with regard to E_1_, free and total E_2_, SHBG, and with regard to the length of each MC and each follicular phase. Nevertheless, the length for 3 MC was extended by isoflavone vs. placebo treatment.

The first RCT of Maskarinec et al. (study A), a double-blind, placebo-controlled trial with parallel group design, provided either 100 mg/d isoflavones by tablets or a placebo (maltodextrine) for 12 months to premenopausal women. Supplementation of isoflavones increased their urinary excretion already after 1 month vs. placebo and remained increased up to the end the study [34,35]. Nevertheless, the serum concentration of E_1_, E_1_S, E_2_, SHBG, FSH, LH, and progesterone remained unchanged. Estrogen metabolites such as 16α-(OH)E_1_, 2-(OH)E_1_, the 2-(OH)E_1_–to-16α-(OH)E_1_-ratio [34,35] and estrone-3-glucuronide [34] were not different after both treatments 1, 3, 6, and 12 months of intervention. Differences in the length of MC [35] and in mammographic parameters [37] were not detectable.

A second study of the same working group (study B), also conducted with premenopausal women in parallel group design, provided 50 mg/d of isoflavones by soy foods. One group ingested two servings of soy foods per day (e.g., tofu, soy milk, roasted soy nuts, soy protein powder or soy protein bars, replacing similar food items) in addition to the usual diet for 24 months. The latter was a low-soy diet (<3 servings of soy food/week) which was used as control [36,38,40,51]. The renal excretion of isoflavones increased [38,40], but changes in the concentration of E_1_, E_2_, free E_2_, FSH, SHBG, and progesterone in serum were not observed [38]. Furthermore, the length of MC [38], mammographic densities [36], IGF-1, IGFBP-3, the IGF-1-to-IGFBP-3-ratio [40], and markers of inflammation (interleukin-6 (IL-6), C-reactive protein (CRP), adiponectin, leptin) [41] were not different between both treatments. When using mixed effect regression models taking either into account randomization group, repeated measurements [45] and additionally ethnicity (Asian vs. non-Asian) [48], the urinary excretion of estrogens (E_1_, E_2_, E_3_) and their metabolites (2-(OH)E_1_, 4-(OH)E_1_, 2-(OH)E_2_, 2-MeOE_1_, 16keto-E_2_, 16α-(OH)E_1_) was comparable throughout the study. The latter model revealed higher serum levels of IGF-1 and IGFBP-3 after high vs. low soy intake [48] than the mixed model for repeated measurements [40]. However, the IGF-1-to-IGFBP-1-ratio was not different between both interventions [40,48]. Breast density, CRP, IL-6, adiponectin, and leptin in serum [41,48], NAF volume, the estrogens’ concentrations in NAF, and the cytological classification of mammary epithelial cells were not different between both treatments [48].

The third RCT of the same working group (study C), a crossover study, provided two servings of soy foods daily for 6 months in addition to a low soy diet. The latter served as control. Both interventions were separated by a 1-month-washout [42,43,44,45,46,47,48]. Again, urinary excretion of isoflavones increased by the consumption of soy foods [43,47,48], but without changes in the estrogens’ concentration in serum [42], NAF [42], and urine [45]. Urinary excretion of most estrogenic metabolites was not different between both treatments except of E_1_S [48], 4-(OH)E_1_ [45], and the 2-(OH)E_1_-to-16α-(OH)E_1_-ratio [45]. NAF volume [43,48] and the cytological classification of mammary epithelial cells from NAF [47] were comparable. The 15-F_2_-isoprostanes-to-creatinine-ratio in urine increased by consumption of soy foods vs. control, also with consideration of the compliance, but significance was failed after excluding participants with very low creatinine levels [46].

### 3.3. Studies with Postmenopausal Women

Six RCTs were performed with postmenopausal women (Table 2). Most were done in parallel group design with an intervention for 2 [54], 10 [53] 12 [49] or 24 [51,52] months. Xu et al. was the only crossover study, with an intervention period of 3 months for each treatment [50]. Daily supplementation of isoflavones in doses of 1 mg/kg BW or 2 mg/kg BW [50], 36.5 mg [49], 80 mg [52], 100 mg [53], and 120 mg [52] for a period of 3 [54], 10 [53], 12 [49], and 24 [52] months by means of soy protein powder [49,50], tablets [51,52], capsules [53], and soy-rich foods [54] increased their concentration in serum [52] and plasma [49,53]. An isoflavone intake of 1.0 mg/kg BW/d and 2.0 mg/kg BW/d with soy protein increased dose-dependently the excretion of several isoflavones in 72-h-urine compared to control treatment (soy protein powder with 0.1 mg isoflavones/kg BW/d) [50].

Hormonal changes in serum/plasma with respect to E_2_, FSH, and LH were not observed after isoflavones’ supplementation [53] and the concentrations were not different after isoflavone and placebo treatment [52]. The renal excretion of E_1_, E_2_, E_3_, total estrogens and estrogen metabolites (2-(OH)E_1_, 4-MeOE_1_, 2-MeOE_1_, 2-(OH)E_2_, 4-(OH)E_2_, 16-ketoE_2_, 17-epiE_3_, 16-epiE_3_) was not different; only 4-(OH)E_1_ was excreted in lower amounts after isoflavone supplementation (1.0 mg/kg BW/d; 2.0 mg/kg BW/d) vs. control treatment (0.1 mg isoflavones/kg BW/d) [50]. Breast density [49,52,53] and the classification of breast parenchyma by the quantity of fibroglandular tissue [53] were not different between isoflavone and control/placebo treatment. For IGF-1, changes in serum or plasma were not detectable between isoflavone and placebo treatment [53] and changes in tumor-necrosis-factor-α, IL-6, adiponectin and resistin were not different 2, 4, 6, and 8 weeks after a soy-rich diet, providing 50 mg/d isoflavones, compared to a soy-free diet with an equal composition of macronutrients [54].

### 3.4. Studies with a Mixed Group of Pre-, Peri- and Postmenopausal Women

Mixed groups of women in pre-, peri-, and postmenopausal state were investigated in four RCTs with either parallel group [55,57,58] or crossover design [56] (Table 3). These were double-blind and placebo-controlled [55,56,57,58] as isoflavones (daily dose: 40 mg [58], 43.5 mg [55], 86 mg [56], 235 mg [57]) were supplied by capsules or tablets for 1 [56], 6 [57], 12 [55], or 36 [58] months. Supplementation of isoflavones increased isoflavone excretion in 24-h-urine after 1 [56], 6, and 12 months [55] and their concentrations in plasma and NAF after 6 months compared to placebo treatment [57].

However, E_2_ [55,57], LH [55], FSH [55,57], SHBG [57] and E_2_/SHBG [57] were not different between both treatments. Changes in breast density [55,58], lymphocytes’ tyrosine kinase activity [55] and in the concentration of IGF-1, IGFBP-1, and IGFBP-3 in serum [56] were not detectable. A correlation between Ki-67 labeling index (Ki-67 LI) and atypical mammary epithelial cells was observed, but changes in Ki-67 LI, atypical cells and in Masood score after 6 months of intervention were not different between isoflavone and placebo treatment [57]. Moreover, in mammary epithelial cells, the changes in the expression of genistein molecular targets genes (BAX, BCL2, BCL3, BIRC5, CCND1, CDKN1A, CDKN2A, DDIT3, FAS, GREB1, NFKB1, PARP-1, PTGS2, TP53), estrogen responsive genes (ESR1, ESR2, FOXA1, IGF1, IGFBP5, MYB, PGR, SCUBE, TFF1), breast epithelial atypia associated genes (PRLR, AR, FGFR3, NDRG2, WNT5B), and housekeeping genes (GAPDH, HPRT1) were not different between both treatments. Changes in NAF volume and in the concentration of E_2_, cathepsin D, epidermal growth factor and IGF-1 in NAF were also comparable [57].

If statistical analysis separated between pre- and postmenopausal women, the same results were obtained as for all women with regard to the changes in the plasma concentration of genistein, equol, E_2_, FSH, progesterone, SHBG, E_2_/SHBG, and the expression of the above mentioned genes from mammary epithelial cells [57].

### 3.5. Risk of Bias Assessment

**Figure 2:**
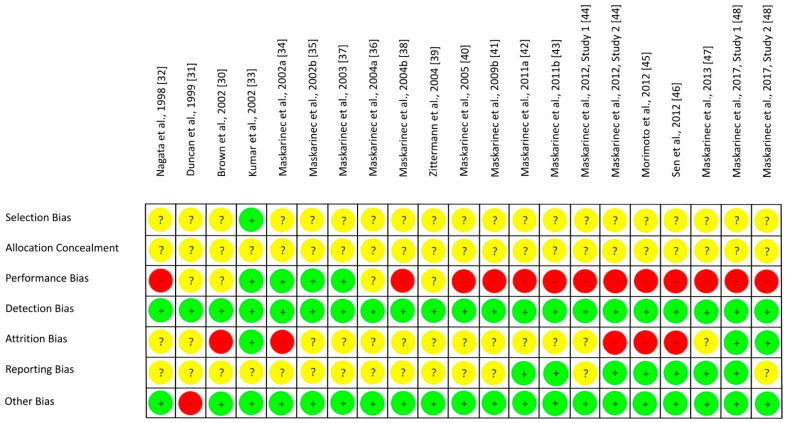
Risk of bias for studies with premenopausal women. Green (+), low risk of bias; yellow (?), unclear risk of bias; red (−), high risk of bias.

**Figure 3:**
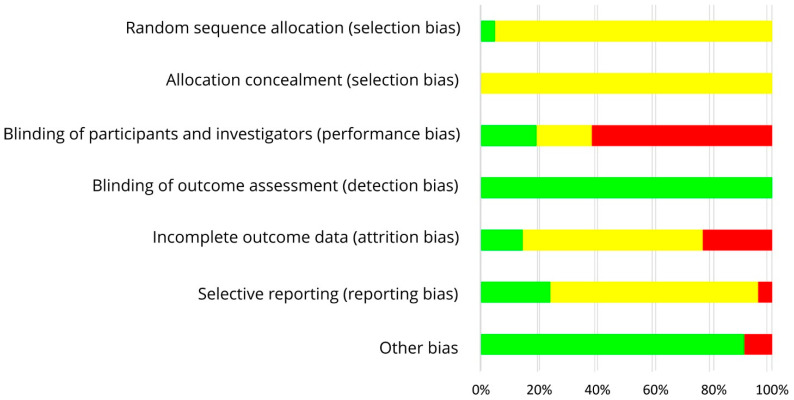
Risk of bias summary across studies with premenopausal women. Green (+), low risk of bias; yellow (?), unclear risk of bias; red (−), high risk of bias.

The results on RoB assessment for individual studies with premenopausal women are shown in Figure 2, based on criteria in Table 4. As most RCTs did not provide any information on the randomization method, risk of selection bias remains unclear [30,31,32,34,35,37,39,40,42,43,44,45,46,47,48]. The RoB for allocation concealment was always unclear as the latter was not considered [30,31,32,33,34,35,37,39,40,42,43,44,45,46,47,48]. If supplements were used for isoflavone treatment, participants and researcher were blinded (low risk of performance bias [33,34,35,37]) or at least the participants (risk bias unclear [30,31]). In studies with soy food, blinding of participants was not possible and remains unclear for researcher (high risk of performance bias [32,36,38,39,40,41,42,43,44,45,46,47,48]). Due to blinding of outcome assessment, the risk of detection bias was always low [30,31,32,33,34,35,37,39,40,42,43,44,45,46,47,48]. Outcome data were mostly incomplete, and dropouts mostly reported for each group/treatment, but not the underlying reasons. For statistical analysis, missing data were only partly considered, leading to a low [33,48], unclear ([31,32,35,36,37,38,39,40,41,42,43,47], Maskarinec et al. 2012, study 1 [44]) or high risk of attrition bias ([30,34,45,46], Maskarinec et al. 2012, study 2 [44]). If the study protocol was registered, outcomes were reported as registered, thus lowering the risk of reporting bias ([42,43,45,46,47], study 1 of Maskarinec et al. 2017 [48]), but registration was often lacking ([30,31,32,33,34,35,36,37,38,39,40,41,44], study 2 of Maskarinec et al. 2017 [48]: unclear risk of reporting bias). For most studies, potential confounder from diet can be excluded as the nutritional behavior was considered and soy food restricted. Compliance with intervention was assessed in each study except of Duncan et al. Thus, other risk of bias was high for Duncan et al. [31] and low for the other studies [30,32,33,34,35,37,39,40,42,43,44,45,46,47,48]. Within studies, overall RoB was unclear [33,35,36,37,39] or high [30,31,32,34,38,40,41,42,43,44,45,46,47,48]. Across RCTs, the risks were low for detection bias and other bias, high for performance bias, and unclear for further bias (Figure 3).

**Figure 4:**
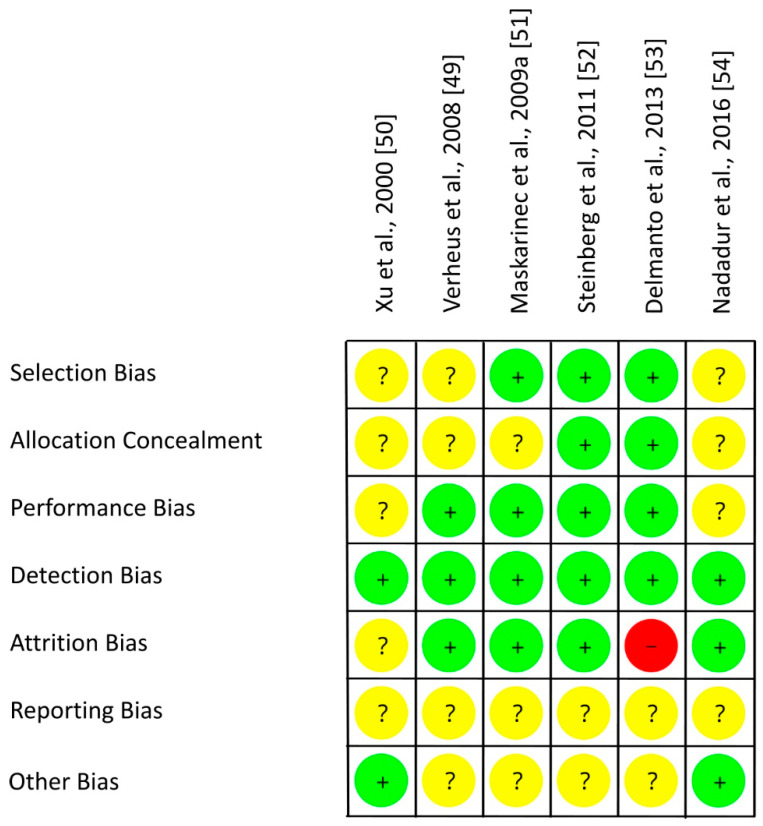
Risk of bias for studies with postmenopausal women. Green (+), low risk of bias; yellow (?), unclear risk of bias; red (−), high risk of bias.

**Figure 5:**
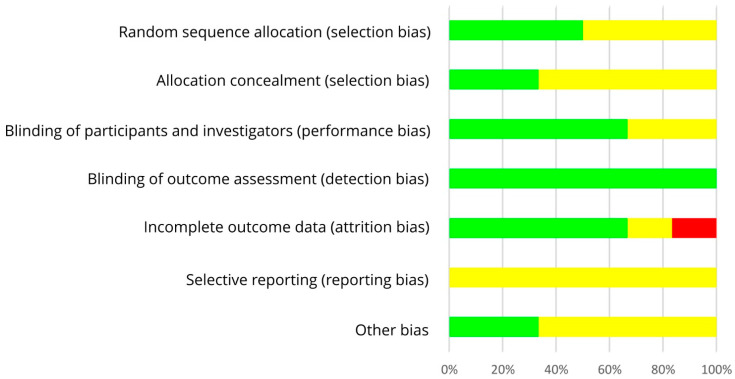
Risk of bias summary across studies with postmenopausal women. Green (+), low risk of bias; yellow (?), unclear risk of bias; red (−), high risk of bias.

As shown in Table 5 and in Figure 4, an adequate randomization method was used by three of six RCTs with postmenopausal women (low risk of selection bias [49,50,54]) which remains unclear for the other three studies (risk of selection bias unclear [51,52,53]). RoB for allocation concealment was low in two studies [52,53], but mostly unclear as allocation concealment was not addressed [49,50,51,54]. The risk of performance bias was low by using supplements which allowed blinding of participants and researchers in most studies [49,51,52,53], but this remains unclear in two RCTs [50,54]. In each study, risk of detection bias was low by blinding outcome assessments [49,50,51,52,53,54]. In case of missing data, number and reasons for dropouts were not reported separately for each treatment and statistical methods to impute missing values were only partly performed. Hence, the risk of attrition bias was low [49,51,52,54], unclear [50] or high [53]. The study protocol was registered for one out of six trials, leading to an unclear risk of reporting bias [49,50,51,52,53,54]. The risk of other bias was low for two [50,54] and unclear for the other four trials [49,51,52,53]. Within trials, overall RoB was mostly unclear [49,51,52,54,59] and in one trial high [53]. Across RCTs, the RoB was low (performance bias, detection bias, attrition bias) or unclear (selection bias, reporting bias, other risk of bias, allocation concealment) (Figure 5).

**Figure 6:**
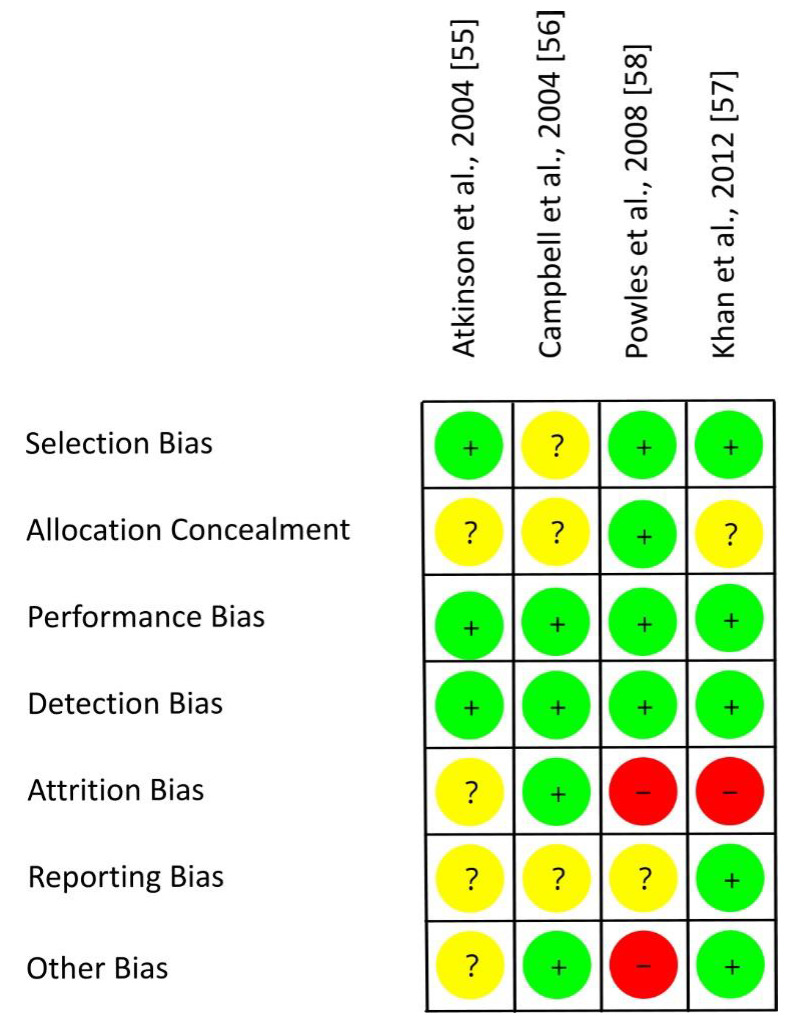
Risk of bias for studies with a mixed group of pre-, peri- and postmenopausal women. Green (+), low risk of bias; yellow (?), unclear risk of bias; red (−), high risk of bias.

**Figure 7:**
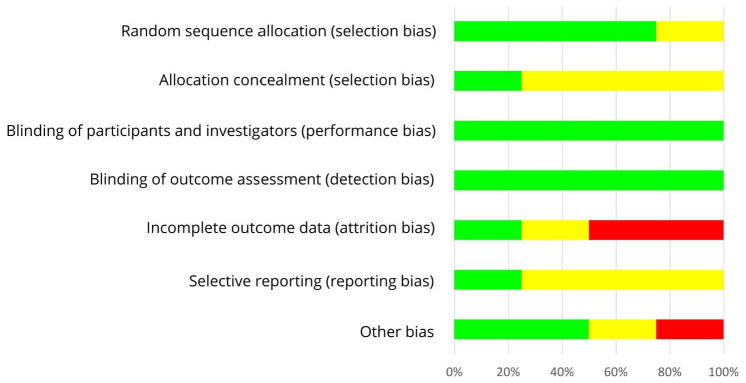
Risk of bias summary across studies with a mixed group of pre-, peri- and postmenopausal women. Green (+), low risk of bias; yellow (?), unclear risk of bias; red (−), high risk of bias.

Figure 6 shows the RoB assessment for individual studies with mixed groups of women on the basis of the criteria presented in Table 6. Three out of four RCTs used an adequate randomization method, thereby reducing the risk of selection bias [55,57,58]. For most studies, the risk of allocation concealment remains unclear [55,56,57]. Each study provided isoflavones by means of tablets and used an isoflavone-free placebo. Due to blinding of participants, researcher and outcomes, the risks of performance bias and detection bias were low for all trials [55,56,57,58]. The risk of attrition bias was low [56], unclear [55], or high [57,58] as dropouts and underlying reasons were not always reported (if reported, not always separately for each group) and statistical methods to impute missing data were only applied in one study. The risk of other bias was different (low [56,57], unclear [55], high [58]) as confounders from diet and adherence to treatment were only partly considered. Within studies, overall RoB was unclear [55,56] or high [57,58]. RoB across RCTs was low for selection bias, performance bias and detection bias, high for attrition bias, and unclear for allocation concealment, reporting bias and other bias (Figure 7).

## 4. Discussion

The aim of this systematic review was to provide an overview on RCTs which investigated the effect of isoflavone intake on risk factors of breast cancer to evaluate the evidence for preventive effects in vivo taking into account the RoB of the studies considered. To the best of our knowledge, this is the first systematic review that provides a detailed picture on potential changes with regard to breast density, estrogen synthesis, estrogen metabolism and biological mechanisms that depend on estrogen response. For this, a variety of mammographic, functional, and laboratory parameters were considered, established risk factors as well as factors being associated with breast cancer risk.

Contrary to our expectations, most RCTs did not find any changes after isoflavone treatment (Table 1, Table 2 and Table 3). However, urinary excretion of isoflavones increased by isoflavone treatment in most trials [34,35,38,39,43,47,48,49,50,52,53,55,56] except of Brown et al. [17] (results not reported by [40,41,42,45,46,54]), suggesting that isoflavones were bioavailable and participants compliant with intervention. Brown et al. [30] provided a low amount of isoflavones (40 mg/d) for a short intervention period (2 MC) compared to RCTs with an increased excretion of isoflavones (50 mg/d for 6 [30,34,35] or 24 months [38,48], 100 mg/d for 12 months [34,35,49,55], 104 mg/d [26] and 86 mg/d [43] for 1 MC, 43.5 mg/d for 12 months [42]).

In studies with premenopausal women, isoflavone treatment does obviously not affect estrogen homeostasis as E_1_ remained unchanged in most [30,33,35,38,39,42,48] and E_2_ in all trials [30,31,32,33,35,38,39,42,43,48]. The concentration of estrogen precursors such as E_1_S [30,31,38,42,48], androstenedione [30,31], progesterone [30,31,35,38,39,48], DHEA [30,31] and DHEAS [30] in serum or plasma did not change either, except of a decrease in DHEAS in the study of Duncan et al. [31]. Changes in the activity of enzymes involved in the synthesis of E_1_ and E_2_, (e.g., 17β-hydroxysteroid dehydrogenase, steroid sulfatase, 3β-hydroxysteroid dehydrogenase) were probably not modulated as shown for isoflavones in vitro [9]. LH and FSH stimulate the synthesis of estrogens in the ovaries by increasing the synthesis of androgens and their conversion to estrogens, respectively [60], but their concentration in serum or plasma did not change during intervention (LH [30,31,35], FSH [31,39]). Isoflavone treatment did not modulate the concentration of SHBG [31,32,33,38,39,48] and the percentage of free E_2_ [33,35,38,39], the active form inducing estrogenic effects [60]. Urinary excretion of 2-(OH)-E_1_ [30,34,44,45,48], 2-(OH)-E_2_ [48], 4-(OH)-E_1_ [48], 4-methoxyestrone (4-MeO-E_1_) [48], and 16α-(OH)-E_1_ [30,34,35,44,45,48] and the concentration of 4-(OH)-E_1_, 16α-(OH)-E_1,_ and 4-MeO-E_1_ in serum were not affected by isoflavone treatment [48]. The ratio of 2-(OH)-E_1_-to-16α-(OH)-E_1_ in urine remained unchanged in most trials [30,34,35,44] except of a decrease in the study of Morimoto et al. [45]. These observations point out that isoflavone treatment does not modulate estrogen homeostasis, the pattern of estrogen metabolites and the amount of active E_2_ in premenopausal women. This, in turn, might explain why the length of MC [30,31,32,33,35,38], breast density [36,37,48], the cytological classification of mammary epithelial cells [47], elements of the insulin-like growth factor (IGF) system [39,48] remained always unchanged even if they are influenced by estrogens. Inflammatory biomarkers like IL-6 [41,48] and CRP ([41], study 1 of Maskarinec et al., 2017 [48]) did not change either, except of a decrease in CRP in study 2 of Maskarinec et al., 2017 [48].

In postmenopausal women, urinary excretion of E_1_ [50], E_1_S [50], E_2_ [50,52,53], progesterone [50], FSH [50,52,53], LH [50,52,53], and SHGB [50] was not modulated by isoflavone treatment. This indicates that isoflavone intake does not affect estrogen homeostasis. For estrogen metabolites, the changes in the concentration of 2-(OH)-E_1_, 2-(OH)-E_2_, 4-(OH)-E_2_, 2-MeO-E_1_, S-MeOE_2_, 16-ketoE_2_, 16-epiE_3_, and 17-epiE_3_ were not different between a diet rich or low in soy food, except of a decrease in 4-(OH)-E_1_ [50]. The increase in the ratio of 2-hydroxylation metabolites to 4-hydroxylation metabolites [2-(OH)-E_1_ + 2-(OH)-E_2_ + 2-MeO-E_1_ + 2-MeO-E_2_] to [4-(OH)-E_1_ + 4-(OH)-E_2_ + 4-(MeO)-E_1_] may be beneficial as 2-OH-metabolites weaken the estrogen effect [59], while 4-OH-metabolites may induce transformation of breast into tumor cells [61]. On the other hand, other ratios of preventive-to-genotoxic metabolites remained unchanged [50], and mammographic parameters [49,51,52,53] as well as biomarkers of inflammation [53,54] were not affected by isoflavone treatment. Interestingly, the mean concentration of genistein and daidzein in serum/plasma after isoflavone treatment reached 144 µM and 220 µM, respectively [53]. Little is known about the concentrations that are needed in vivo to reduce the activity of enzymes of 2- and 4-hydroxylation pathway. As reported recently, an inhibition of cytochrome P450 1B1 by 50% lowers the production of 4-OH metabolites from recombinant cells by 3 µM genistein. However, genistein occurs in vivo mainly as metabolite due to intestinal conjugation, and not in free form. If breast cells react similarly to recombinant cells remains unclear. Unfortunately, data on IC_50_-values for daidzein and cytochrome P450 1A1 are not available yet [9].

In women with different menopausal states, changes in estrogen homeostasis by isoflavone treatment are also unlikely as the serum/plasma concentration of E_2_ [55,57], progesterone [57], FSH [55,57,58], LH [55], SHBG [57], and SHBG/E_2_ [57] remained unchanged. Parameters influenced by estrogens (breast density [55,58], growth factors [56], cytological classification of mammary epithelial cells [57], components of NAF [57]) were not different between both treatments although 10-times higher concentrations of genistein in NAF were achieved by isoflavone vs. placebo treatment [57]. Differences in the expression of genes related to proliferation, apoptosis and other estrogenic effects were not detectable between isoflavone and placebo group [57]. Therefore, isoflavone supplementation even in a large dose of 235 mg/d for 6 months does not modulate the expression of genes involved in the regulation of proliferation, apoptosis, and inflammation. If data were analyzed separately for pre- and postmenopausal women, differences between the subgroups were not detectable either [56,57]. It is well known that both, the estrogen concentration in serum/plasma and the expression of ER-β, are reduced in post- compared to premenopausal women. This in turn enhances proliferative and inflammatory response, thereby increasing the risk of breast cancer [62]. With regard to the mechanisms of isoflavones on estrogen synthesis, metabolism and estrogen response, effects by isoflavone intake were especially expected in postmenopausal women but were not found.

Taken together, most RCTs with pre- and/or postmenopausal women did not show a response to isoflavone treatment, although the dose of isoflavones ingested and the duration of treatment varied between the studies (premenopausal: 40 mg/d up to 2 mg/kg BW/d for 1–24 months; postmenopausal: 36.5 mg/d up to 2 mg/kg BW/d for 2-24 months; mixed groups: 40 mg/d up to 235 mg/d for 1–36 months). Breast density was determined after 10 [53], 12 [37,49,55], 24 [36,48,51,52] and 36 [58] months as changes in breast density afford more time than changes in laboratory and functional parameters. Whether the response to isoflavone treatment differs between geographical regions, as suggested from some epidemiological studies in Asia and Western countries [6,7], remains unclear as a single RCT was performed in Japan [32] and most results were obtained from RCTs in Western Europe [39,49,55,56,58] and USA [33,34,36,37,38,40,41,42,43,44,45,46,47,48,50,51,52,54,57].

The overall RoB in trials with premenopausal women was often higher (16 × high, 5 × unclear) than in trials with postmenopausal women (1 × high, 5 × unclear) and in mixed groups (2 × high, 2 × unclear). The risk of attrition bias was quite different between the studies. For most studies, the risk of selection bias, reporting bias and the risk of allocation concealment remains unclear as relevant details were not reported, and the study protocol not registered in clinicaltrials.gov. The risk of detection bias was always, and the risk of other bias often low. As the overall RoB of RCTs funded by industry was high [34,53] or unclear [35,55], similar to RCTs without industrial funding (high RoB [30,31,32,38,40,41,42,43,44,45,46,47,48,57,58], unclear RoB [33,35,36,37,39,49,50,51,52,54,56]), and the results comparable, industrial funding as further source of bias seems to be rather unlikely.

The sample size was calculated in four trials [53,55,57,58], but in two RCTs for vasomotor symptoms [53] and bone density [58] being not relevant for this review. The other used the Wolfe pattern [55] and Ki-67 LI [57] for sample size calculation. The number of cases included in the statistical evaluation of both trials was above the calculated sample size. Hence, the lack of changes in Wolfe pattern [55] and Ki-67 LI [57] (primary outcome markers) clearly suggests that isoflavone treatment does not modulate these parameters. However, for trials without sample size calculation which did not find statistically significant effects, it remains open if there is no effect or if this is not detectable [30,31,32,33,34,35,36,37,38,39,40,41,42,43,44,45,46,48,49,50,51,52,53,54,56,58].

Moreover, pooling data as in meta-analyses increases the sample size and the probability to detect an effect by isoflavone treatment. A meta-analysis of eight RCTs published in 2010 investigated the impact of isoflavone-rich foods or supplements on breast density and related parameters. A small increase in breast density was detectable for premenopausal women, but not for postmenopausal women and all women.

This systematic review of RCTs investigates the response of isoflavone treatment to parameters which are considered as risk factor for breast cancer. These RCTs were described in detail and assessed for RoB to provide a clear picture on the effect induced by isoflavone intake in women with different menopausal states. Moreover, each study was checked for sample size calculation to evaluate imprecision.

Literature was only searched in PubMed as this has shown to be an optimal tool in the field of biomedical research, even with free access [27,28]. With regard to the results of previous investigations [27,28], an additional search in Google Scholar and in paid databases such as Scopus and Web of Science was unlikely to provide further records of relevance for this review. However, this remains speculative as records from other databases were not available for comparison. Thus, the restriction to literature search in PubMed might be a limitation.

## 5. Conclusions

Risk factors of breast cancer (breast density, estrogens and estrogen metabolites and further parameters related to estrogen response) did not change in most trials despite a good adherence to isoflavone treatment, independent of the kind of intervention, the dose of isoflavones used and the duration of isoflavone treatment. However, the lack of significant changes does not prove the lack of effects as a sample size calculation was missing in most studies. Taking into account the RoB and methodological limitations, there is little evidence that isoflavone treatment modulates risk factors of breast cancer in pre- and postmenopausal women.

Future studies should calculate the samples size based on existing results to allow clear conclusions and should report further methodological details to reduce RoB. A meta-analysis of RCTs is warranted to judge if an isoflavone intake might contribute to the prevention of breast cancer.

## Figures and Tables

**Table 1:** Effects of isoflavone intake in premenopausal women on parameters related to the risk of breast cancer.

Study, Ref., Country	Participants	*n* ^1^	Study Design	Intervention/d, Isoflavone Intake/d	Duration (I)	Parameter	Results
Nagata et al., 1998, [32], Japan	EX: pregnancy, hormone preparations, endocrine disorders	60	Parallel group	Form: soy food I: soy milk, 400 mL (109 mg isoflavones: 3 mg daidzein, 38 mg daidzin, 3 mg genistein, 65 mg genistin) C: regular diet	3 MC	E_1_ (Serum: d11 of MC 1 and 3)	↓ I, ∅ C I < C (MC3)
						E_2_, SHBG (Serum: d11 of MC 1 and 3)	ΔI = ΔC
						Length of MC (Considering the following 2 MC)	ΔI = ΔC
Duncan et al., 1999, [31], USA	EX: pregnancy, breastfeeding, irregular MC, smoking, antibiotics, or hormones ≤6 m, history of chronic disorders including endocrine or gynecological diseases, benign breast disease, regular medication including aspirin, <90% or >120% ideal BW, change in BW >10 lb ≤1 y or >5 lb ≤2 m, vegetarian, high fiber/soy or low-fat diets, regular supplementation of micronutrients > RDA, athleticism, >2 alcoholic beverages/d, history of food allergy	14	CR, 3 wk washout	Form: soy protein powder with different isoflavone content: I_1_: 1.01 mg/kg BW; I_2_: 2.01 mg/kg BW; C: 0.15 mg/kg BW (55% genistein, 37% daidzein, 8% glycitein)	3 MC + 9d	E_1_ (Plasma: d7 after LH surge in MC2 until end of each intervention)	I_2_ < I_1_ (MF); I_1_ = I_2_ = C (EF, PO, ML)
						LH, FSH (Plasma: d7 after LH surge in MC2 until end of each intervention)	I_1_ < C (PO); I_1_ = I_2_ = C (EF, MF, ML)
						E_1_S, P (Plasma: d7 after LH surge in MC2 until end of each intervention)	I_1_ = I_2_ = C (EF, MF, PO, ML)
						T, A4, DHEA, SHBG, P (Plasma: early follicular phase (d2–5) of MC3 and MC4)	I_1_ = I_2_ = C
						DHEAS (Plasma: early follicular phase (d2–5) of MC3 and MC4)	I_2_ < I_1_
						Length of MC, follicular phase, and luteal phase (MC2 and MC3; ovulation according to predictor kit)	I_1_ = I_2_ = C
Brown et al., 2002, [30], USA	EX: pregnancy, breastfeeding, OC ≤ 6 m, irregular MC, antibiotics ≤3 m, history of chronic disorders, >2 alcoholic drinks/d, >25 g fiber/d, >2 serv. of soy foods/wk, vegetarian, smoker, <90% or >120% ideal BW, strong changes in BW, food allergy	14	CR, single-blind, 2 wk washout	Form: supplements I: 31 g soy protein (40 mg isoflavones: 26 mg genistein, 11 mg daidzein, 3 mg glycitein) in addition to a high-fat Western diet C: high-fat Western diet without soy protein	2 MC	E_1,_ E_2_, E_1_S, P, T, A4, DHEA, DHEAS, SHBG, PRL (mid-follicular and mid-luteal phase), FSH (mid-follicular phase), LH (mid-luteal phase) (Serum: d7 and d8, or d8 and 9d after menses (mid-follicular phase) and d21 and d22 or d22 and d23 after menses (mid-luteal phase))	I = C
						2-(OH)E_1_, 16α-(OH)E_1_, 2-(OH)E_1_-to-16α-(OH)E_1_-ratio (48-h-Urine: pooled urine of the same days as for serum collection)	I = C
						Length of MC (Ovulation kit, body temperature)	I = C
						Isoflavones (daidzein, genistein, equol, *O*-DMA; sum of all) (48-h-Urine: pooled urine of the same days as for serum collection)	I = C
Kumar et al., 2002, [33], USA	EX: pregnancy, breastfeeding ≤ 12 m, irregular MC, hormone preparations, antibiotics ≤3 m, history of cancer, BMI > 38 kg/m², <20 g fiber/d or fiber supplementation, consumption of soy products, soy or casein allergy, vegan	66	Parallel group, double-blind, placebo-controlled	Form: supplements I: soy protein (40 mg isoflavones as genistein) C: milk protein as placebo	3 MC	E_1_, E_2_ (free, total), SHBG (Serum: 0, 3 MC; always 3 d after onset of menstruation)	ΔI = ΔC
						Length of MC and follicular phase (MC1, MC2, MC3, FC1, FC2, FC3; determined from days of menses, ovulation and absence of menses, and of ovulation)	I = C (MC1, MC2, MC3, FC1, FC2, FC3, FC1-FC3); I > C (MC1-MC3)
Maskarinec et al., 2002a, [34], USA, Study A	EX: hormone preparations, intended pregnancy, no intact uterus/ovaries, irregular MC, serious medical conditions, history of cancer	28	Parallel group, double-blind, placebo-controlled, 2 wk run-in	Form: tablets I: 100 mg isoflavones C: placebo	12 m	Isoflavones (Σ daidzein, genistein, glycetin, equol, *O*-DMA) (Urine: 0, 1, 3, 6, 12 m; ~5d after ovulation)	I > C (1, 3, 6, 12 m)
						E_1_-3-G, 16α-(OH)E_1_, 2-(OH)E_1_, 2-(OH)E_1_-to-16α-(OH)E_1_-ratio; adjusted for creatinine excretion (Urine: time of sampling: see above)	I = C (1, 3, 6, 12 m)
Maskarinec et al., 2002b, [35], USA, Study A	EX: hormone preparations, intended pregnancy, no intact uterus/ovaries, irregular MC, serious medical conditions, history of cancer, >7 serv. of soy foods/wk	28	Parallel group, double-blind, placebo-controlled, 2 wk run-in	Form: tablets I: 100 mg isoflavones (51% daidzein, 44% genistein, 5% glycitein) C: maltodextrine as placebo	12 m	Isoflavones (Σ daidzein, genistein, glycetin, equol, *O*-DMA) (Urine: 0, 1, 3, 6, 12 m; ~5 d after ovulation)	I > C (1, 3, 6, 12 m)
						E_1_, E_1_S, E_2_, Free E_2_, SHBG, FSH, LH, P, 16α-(OH)E_1_, 2-(OH)E_1_, 2-(OH)E_1_-to-16α-(OH)E_1_-ratio (Urine: time of sampling: see above)	I = C (1, 3, 6, 12 m)
						Length of MC (Ovulation kit, MC calendar; 1, 2–3, 4–6, 6–12 m)	I = C (1, 2-3, 4–6, 6–12 m)
Maskarinec et al., 2003, [37], USA, Study A	EX: OC <3 m, hormone preparations, intended pregnancy ≤1 y, no intact uterus/ovaries, irregular MC, no normal mammogram ≤6 m, serious medical conditions, history of cancer, >7 serv. of soy foods/wk	30	Parallel group, double blind, placebo-controlled	Form: tablets I: 100 mg isoflavones (51% daidzein, 44% genistein, 5% glycitein) C: maltodextrine as placebo	12 m	Total breast area, dense area, breast density (%) (Mammography: 0, 12 m)	ΔI = ΔC
Maskarinec et al., 2004a, [36], USA, Study B	EX: OC, hormone preparations, no uterus/intact ovaries, irregular MC, abnormal screening mammography, history of cancer, ≥6 serv. of soy foods/wk	201	Parallel group	Form: soy foods I: soy-rich diet (2 serv. of soy foods: tofu, soy milk, roasted soy nuts, soy protein powder, or soy protein bars; 50 mg isoflavones) C: low-soy diet (regular diet)	24 m	Total breast area, dense area, breast density (%) (Mammography: 0, 24 m)	ΔI = ΔC
Maskarinec et al., 2004b, [38], USA, Study B	EX: OC or any hormone preparations, no uterus/ovaries, irregular MC, previous history of cancer, ≥6 serv. of soy foods/wk	189	Parallel group	Form: Soy foods I: soy-rich diet (2 serv. of soy foods: tofu, soy milk, roasted soy nuts, soy protein powder, or soy bars; 50 mg isoflavones) C: low-soy diet (regular diet)	24 m	Isoflavones^2^ (Urine: 3, 6, 12, 24 m; always 5d after ovulation)	I > C
						E_1,_ E_2_, free E_2_, E_1_S, SHBG, P (Serum: time of sampling: see urine)	ΔI = ΔC
						Length of MC (Ovulation kit)	I = C
Zitter-mann et al., 2004, [39], Germany	EX: pregnancy, OC, irregular MC, amenorrhea, chronic diseases, eating disorders, BMI <18 kg/m^2^, non-Caucasian	14	CR, placebo-controlled, 2 MC washout	Form: soy foods I: 5 soy cookies (52 mg isoflavones: 19 mg daidzein, 33 mg genistein) C: 5 soy-free cookies with white flour as placebo	1 MC	Daidzein, genistein (Urine: 3 d after onset of menstruation, 3 d before ovulation, midluteal phase, 3 d after onset of next menstruation)	I > C
						E_1_, E_2_, free E_2_, FSH, SHBG, P (Serum: time of sampling: see urine)	I = C
Maskarinec et al., 2005, [40], USA, Study B	EX: hormone preparations, no uterus, no ovaries, irregular MC, cancer, >7 serv. of soy foods/wk	196	Parallel group	Form: soy foods I: soy-rich diet (2 serv. of soy foods, replacing similar food items; 50 mg isoflavones) C: low soy diet (usual diet, <3 serv. of soy foods/wk)	24 m	Isoflavones: genistein, daidzein, dihydrogenistein, glycitein, dihydrodaidzein, *O*-DMA, equol (Urine: 0, 3, 6, 12, 24 m; 19 d of ovulation cycle/ 5 d after ovulation)	n.d.
						IGF-1, IGFBP-3, IGF-1-to-IGFBP-3-ratio ^3^ (Serum: 0, 3, 6, 12, 24 m; 19 d of ovulation cycle/ 5 d after ovulation)	I = C
Maskarinec et al., 2009b, [41], USA, Study B	EX: OC, hormone preparations, no intact ovaries, hysterectomy, irregular MC, breast cancer	183	Parallel group	Form: soy foods I: soy-rich diet (2 serv. of soy foods, replacing similar food items; 50 mg isoflavones) C: low soy diet (usual diet with <3 serv. of soy foods/wk)	24 m	Isoflavones ^2^ (Urine)	n.d.
						IL-6, CRP, adiponectin, leptin (Serum: 0, 3, 6, 12, 24 m)	I = C
Maskarinec et al., 2011a, [42], USA, Study C	IN: ≥10 μL NAF EX: OC, pregnancy, breastfeeding, no uterus/ovaries, irregular MC, breast implants, previous diagnosis of cancer, >5 serv. of soy foods/wk	82	CR, 1 m washout	Form: soy foods I: soy-rich diet (2 serv. of soy foods, replacing similar food items; 50 mg isoflavones) C: low soy diet (usual diet, <3 serv. of soy foods/wk)	6 m	Daidzein, genistein, equol, *O*-DMA (Urine: 0, 3, 6 m, ~5 d after ovulation)	Data not shown except for equol at baseline (52% equol producer)
						E_1_, E_2_, E_1_S (Serum: 0, 6 m)	ΔI = ΔC
						E_2_, E_1_S (NAF: 0, 6 m)	ΔI = ΔC
Maskarinec et al., 2011b, [43], USA, Study C	IN: ≥10 μL NAF EX: OC, pregnancy, breastfeeding, no uterus, irregular MC, breast implants, isoflavone supplements, previous cancer diagnosis, >5 serv. of soy foods/wk	82	CR, 1 m washout	Form: soy foods I: soy-rich diet (2 serv. of soy foods (soy milk, tofu, or soy nut); 50 mg isoflavones C: low soy diet (usual diet, <3 serv. of soy foods/wk)	6 m	Isoflavones (Σ daidzein, genistein, *O*-DMA, equol) (Urine: 0, 1, 3, 6 m, ~5 d after ovulation)	I > C
						NAF volume (NAF: 0, 3, 6 m, ~5 d after ovulation)	I = C
Maskarinec et al., 2012, [44], USA, S1: Study B S2: Study C	EX: OC, pregnancy, breastfeeding, irregular MC, hysterectomy, breast implants, cancer, supplements of isoflavones, <5 serv. of soy foods/wk	S1: 188 S2: 79	S1: Parallel group S2: CR, 1 m washout	Form: soy foods I: 2 serv. of soy foods; 50 mg isoflavones C: <3 serv. of soy foods/wk	S1: 24 m S2: 6 m	E_1_, E_2_, E_3_, 2-(OH)E_1_, 2-(OH)E_2_, 2-MeOE_1_, 16keto-E_2_, 16α-(OH)E_1;_ each related to creatinine	ΔI = ΔC ^c^ (S1, S2)
						4-(OH)E_1/_creatinine	ΔI = ΔC ^c^ (S1) I < C (S2)
						2-(OH)E_1_-to-16α-(OH)E_1_-ratio	ΔI = ΔC ^c^ S1, S2
						(Urine, S1: 0, 24 m; end luteal phase Urine, S2: 0, 6, 13 m luteal phase)	
						Equol producer (Urine, S1: 0, 24; end luteal phase Urine, S2: 0, 6, 13 m; luteal phase)	S1: *n* = 23, I = C, 12% S2: *n* = 41, 52%
Morimoto et al., 2012, [45], USA, Study C	IN: ≥10 μL NAF EX: estrogen-containing OC, pregnancy, breastfeeding, irregular MC, no uterus, breast implants, cancer, >5 serv. of soy foods/wk	82	CR, 1 m washout	Form: soy foods I: 2 serv. of soy foods; 50 mg isoflavones C: <3 serv. of soy foods/wk	6 m	Isoflavones: daidzein, genistein, *O*-DMA, equol equol producer, non-equol producer (Urine: 0, 3, 5, 7, 8, 10, 12 m)	n.d. (*n* = 43/*n* = 39)
						E_1_, E_2_, E_3,_ 2-(OH)E_1_ (Urine: 0, 3, 5, 7, 8, 10, 12 m)	ΔI = ΔC
						16α-(OH)E_1_ (Urine: 0, 3, 5, 7, 8, 10, 12 m)	I = C
						2-(OH)E_1_-to-16α-(OH)E_1_-ratio (Urine: 0, 3, 5, 7, 8, 10, 12 m)	I > C
Sen et al., 2012, [46], USA, Study C	EX: OC, pregnancy, breastfeeding, irregular MC, breast implants, hysterectomy, cancer, isoflavone supplementation, >5 serv. of soy foods/wk	82	CR, double-blind, 1 m washout	Form: soy foods I: soy-rich diet (2 serv. of soy foods replacing similar food items; 50 mg isoflavones) C: low soy diet (usual diet, <3 serv. of soy foods/wk)	6 m	Isoflavones: daidzein, genistein equol producer (Urine: 0, 6, 13 m)	n.d. *n*=43; 52%
						Excluding subjects with low creatinine values during intervention	I = C
						Woman + Compliance	I > C
						15-F_2t_-IsoP/creatinine (Urine: 0, 6, 13 m)	I > C (all)
Maskarinec et al., 2013, [47], USA, Study C	IN: ≥10 µL NAF EX: OC, pregnancy, breastfeeding, irregular MC, breast implants, hysterectomy, cancer, isoflavone supplementation, <5 serv. of soy foods/wk	82		Form: soy foods I: soy-rich diet (2 serv. of soy foods; 50 mg isoflavones) C: low-soy diet (<3 serv. of soy foods/wk)	6 m	Isoflavones ^2^ (Urine: 0, 6 m)	I > C (6 m)
						Mammary epithelial cells, cytological classification (benign, atypical, malignant); subclassification (normal cells, hyperplasia, single atypical cells, papillary cluster of atypical cells, malignant cells) (NAF: 0, 6 m)	NAF (*n* = 33) ΔI = ΔC
Maskarinec et al., 2017, [48], USA, S1: Study B S2: Study C	EX: OC, pregnancy, breastfeeding, irregular MC, breast implants, hysterectomy, history of cancer, isoflavone supplementation, >5 serv. of soy foods/wk	S1: 189 S2: 82	S1: Parallel group; S2: CR, 1 m washout	Form: soy foods I: soy-rich diet (2 serv. of soy foods, replacing similar food items; 50 mg isoflavones) C: low soy diet (usual diet, <3 serv. of soy foods/wk)	S1: 24 m S2: 6 m	Isoflavones ^2^ equol (Urine, S1: 0, 24 m Urine, S2: 0, 6, 13 m Both studies: ~5 d after ovulation)	I > C ^4^ (S1, S2) I > C ^4^ (S2)
						E_1_, E_2_, 2-(OH)E_1_, 2-(OH)E_2_, E_1_S, 2-MeOE_1_, 4-(OH)E_1_, E_3_, 16-keto E_2_, 16α-(OH)E_1_, SHBG, P (Serum, urine: S1: 0, 24 m; ~5 d after ovulation; S2: 0, 6, 13 m; ~5 d after ovulation)	ΔI = ΔC ^4^ (S1, S2)
						E_1_S (NAF, S2: 0, 6 or 7, 10 or 13 m)	I < C ^4^ (S2)
						IGF-1, IGFBP-3 (Serum, S1: 0, 24 m, ~5 d after ovulation)	ΔI = ΔC ^4^ (S1)
						IGF-1-to-IGFBP-3 ratio (Serum, S1: 0, 24 m, ~5 d after ovulation)	I = C ^4^ (S1)
						CRP, IL-6, adiponectin, leptin (Serum, S1: 0, 24 m, ~5 d after ovulation)	I = C ^4^ (S1), I < C (S1, CRP)
						NAF volume (NAF, S2: 0, 6 or 7, 10 or 13 m)	ΔI = ΔC ^4^ (S2)
						Breast density (%) (Mammography, S1: 0, 24 m)	ΔI = ΔC ^4^ (S1)

↓ significant decrease; ∅ no significant change; I = C: no significant difference between intervention and control treatment; ΔI = ΔC: changes not significantly different between intervention and control treatment; no treatment effect. E_1_-3-G: estrone-3-glucuronide; 16α-(OH)E_1_: 16α-hydroxy-estrone; 16-epiE_3_: 16-epi-estriol; 17-epiE_3_: 17-epi-estriol; 16-ketoE_2_: 16-keto-estradiol; 2-(OH)E_1_: 2-hydroxy-estrone; 2-(OH)E_1_/16α-(OH)E_1_-ratio: 2-hydroxy-estrone to 16α-hydroxy-estrone-ratio; 2-(OH)E_2_: 2-hydroxy-estradiol; 2-MeOE_1_: 2-methoxy-estrone; 2-MeOE_2_: 2-methoxy-estradiol; 2-total:4-total: [2-hydroxy-estradiol + 2-methoxy-estradiol] to 4-hydroxy-estradiol ratio; 2E_1_-total:4E_1_-total: [2-hydroxy-estrone + 2-methoxy-estrone] to [4-hydroxy-estrone + 4-methoxy-estrone] ratio; 4-(OH)E_1_: 4-hydroxy-estrone; 4-(OH)E_2_: 4-hydroxy-estradiol; 4-MeOE_1_: 4-methoxy-estrone; 4-MeOE_2_: 4-methoxy-estradiol; 15-F_2t_-IsoP: 15-F_2t_-isoprostane; DHEA: dehydroepiandrosterone; DHEA-S: dehydroepiandrosterone sulfate; E_1_: estrone; E_1_S: estrone sulfate; E_2_: estradiol; E_3_: estriol. A4: androstenedione; BW: body weight; C: control; CR: crossover; CRP: C-reactive protein; d: day(s); EX: exclusion criteria; FSH: follicle-stimulating hormone; HRT: hormone replacement therapy; I: intervention; IGF-1: insulin-like growth factor 1; IGFBP-1: insulin-like growth factor binding protein 1; IGFBP-3: insulin-like growth factor binding protein 3; IL-6: interleukin 6; IN: inclusion criteria; LH: luteinizing hormone; m: month(s); MC: menstrual cycle; MF: midfollicular phase; ML: midluteal phase; NAF: nipple aspirate fluid; n.d.: no data available; OC: oral contraceptive; *O*-DMA: *O*-desmethylangolensin; PO phase: periovulatory phase; P: progesterone; PRL: prolactin; rFNA: random fine needle aspiration; RDA: recommended dietary allowances; serv.: servings; S: study; SHBG: sex hormone binding globulin; T: testosterone; TNF-α: tumor necrosis factor α; wk: week(s); y: year(s). ^1^ Participants who finished the study and for whom results were available, otherwise, participants who were randomized; ^2^ unclear which isoflavones were measured; ^3^ analysis by mixed-effects regression model taking into account randomization group and repeated measurements; ^4^ mixed-effects regression analysis also considering ethnicity (Asian vs. non-Asian) as a fixed effect. Study A, Study B, Study C in the first column indicate which publications derived from the same study.

**Table 2:** Effects of isoflavones intake in postmenopausal women on parameters related to the risk of breast cancer.

Study, Ref., Country	Participants	*n* ^1^	Study Design	Intervention/d; Isoflavone Intake/d	Duration (I)	Parameter	Results
Xu et al., 2000, [50], USA	EX: regular medication including aspirin, hormones, or antibiotics ≤6 m, menstruation ≤12 m, hysterectomy, oophorectomy, FSH <25 IU/l, history of chronic disorders including endocrine or gynecological diseases, benign breast disease, <90% or >120% ideal BW, weight change >10 pounds ≤1 y, smoking, athleticism, micronutrient supplementation >RDA, inability to abstain from alcoholic beverages during study, strict vegetarian/high fiber/high soy/low fat diet	18	CR, 3 wk washout	Form: soy protein powder providing different amounts of isoflavones I_1_: 1.00 ± 0.01 mg/kg/BW I_2_: 2.00 ± 0.02 mg/kg/BW C: 0.11 ± 0.01 mg/kg/BW (isoflavone pattern: 58% genistein, 33% daidzein, 9% glycitein)	93 d	Genistein, daidzein, glycitein, equol, *O*-DMA, dihydrodaidzein, coumesterol (72-h-pooled urine: before and after each intervention, ~5 d after ovulation)	Total and most individual isoflavones: I_2_ > I_1_ > C
						E_1_, E_1_S, E_2_, SHGB, FSH, LH, P, 16α-(OH)E_1_, 2-(OH)E_1_, 2-(OH)E_2_, 4-(OH)E_1_, 4-(OH)E_2_, 2-MeOE_1_, 2-MeOE_2_, 16-ketoE_2_, 16-epiE_3_, 17-epiE_3_, Genotoxic: total; 2-(OH)E_1_:16α-(OH)E_1_, 2E_1_-total:16α-total, 2E_1_-total:4E_1_-total, 2E_2_-total:4E_2_-total, 2-total:4-total (72-h-pooled urine: before and after each intervention, ~5 d after ovulation)	Total estrogens, individual metabolites, estrogen metabolite ratios: I_2_ = I_1_ = C Except for: 4-(OH)-E_1_: I_1/2_ < C; 2E_1_-total:4E_1_-total: I _1_ > C
Verheus et al., 2008, [49], NL	IN: age 60–75 y EX: HRT < 6 m, active liver or renal disease, history of thromboembolism, former/present malignancy (except of non-melanoma skin cancer), endometrium thickness > 4 mm, lactose intolerance, milk or soy allergy	126	Parallel group, double-blind, placebo-controlled	Form: soy powder I: 36.5 g soy powder providing 99 mg isoflavones (52 mg genistein, 41 mg daidzein, 6 mg glycitein) enriched with vitamins and minerals C: 36.5 g milk protein powder enriched with vitamins and minerals as placebo	12 m	Genistein Equol (Plasma: 12 m)	I > C n.d.
						Breast density (absolute, density %, non-dense area) (Mammography: 0, 12 m)	ΔI = ΔC, no differences for equol vs. non-equol producer
Maskarinec et al., 2009a, [51], USA	IN: age 40–60 y, FSH > 30 IU/L EX: HRT, osteoporosis, spine/hip fracture, cancer, liver, kidney, gallbladder/heart disease, favor bone loss or disease criteria, smoking or former smoking < 5 y, high physical activity, completely sedentary, BMI ≥ 30 kg/m², soy allergy, supplementation, vegetarian, ≥1 serving of soy/wk	325	Parallel group, double-blind, placebo-controlled, multicenter study	Form: tablets I_1_: 80 mg Isoflavones I_2_: 120 mg Isoflavones I_1_, I_2_: 1% genistein, 2% daidzein, 42% daidzin, 13% genistin, 3% glycitein, 39% glycitin C: placebo	24 m	Breast area, dense area, non-dense area, density (%) (Mammography: 0, 12, 24 m)	ΔI_1_ = ΔI_2_ = ΔC
Steinberg et al., 2011, [52], USA	IN: age 40–60 y, FSH >30 IU/mL, ≥12 m of amenorrhea EX: abnormal result from screening mammogram, Papanicolau or blood chemistry test, BMI > 30 kg/m^2^, smoking, history of osteoporosis, spine/hip fracture, cancer, active liver, kidney, gallbladder/heart disease, osteopenia	362	Parallel group, double-blind, placebo-controlled	Form: tablets I_1_: 80 mg Isoflavones (9.9 mg genistein, 44.0 mg daidzein, 27 mg glycitein) I_2_: 120 mg Isoflavones (14.9 mg genistein, 66.3 mg daidzein, 40.6 mg glycitein) C: placebo All ingested additionally a multivitamin preparation providing vitamin D and Ca	24 m	Genistein, daidzein, glycetin Equol producer (Serum: 0, 12, 24 m)	ΔI_2_ > ΔI_1_ > ΔC 33%
						LH, FSH, E_2_ (Serum: 0, 12, 24 m)	I_2_ = I_1_ = C (0, 12, 24 m)
						Breast density, presence/absence of lesions ^2^ (Mammography: 0, 12, 24 m)	I_2_ = I_1_ = C (0, 12, 24 m)
Delmanto et al., 2013, [53], Brazil	IN: >12 m amenorrhea, vasomotor symptoms ≥ 5/d EX: history of cancer, chronic diseases, chronic alcoholism, breast reduction, vegetarian, high intake of fiber or soy	66	Parallel group, double-blind, placebo-controlled	Form: capsule I: soy extract; 100 mg isoflavones (50 mg genistein, 35 mg daidzein) C: lactose as placebo	10 m	Genistein, daidzein (Plasma: 10 m)	I > C
						FSH, LH, E_2_ (Serum/plasma: 0, 10 m)	ΔI = ΔC
						IGF-1 (Serum/plasma: 0, 10 m)	ΔI = ΔC
						Breast density (Mammography: 0, 10 m)	I = C (0, 10 m)
						Breast parenchyma (Ultrasound: 0, 10 m)	I = C (0, 10 m)
Nadadur et al., 2016, [54], USA	IN: age ≥50 y EX: HRT ≤ 6 m, history of cancer (except of non-melanoma skin cancer), diabetes mellitus, other chronic illness, low fat or high fibre diet	37	Parallel group, single-blind	Form: soy foods I: soy rich diet with 15 g soy protein providing 50 mg isoflavones C: balanced diet without soy food, with equal composition of macronutrients	2 m	Isoflavones^3^ (Urine: 0, 2, 4, 6, 8 w)	n.d.
						TNF-α, IL-6, adiponectin, resistin (Serum: 0, 2, 4, 6, 8 w)	ΔI = ΔC

ΔI = ΔC: changes not significantly different between intervention and control treatment (no treatment effect). 16α-(OH)E_1_: 16α-hydroxy-estrone; 16-epiE_3_: 16-epi-estriol; 17-epiE_3_: 17-epi-estriol; 16-ketoE_2_: 16-keto-estradiol; 2-(OH)E_1_: 2-hydroxy-estrone; 2-(OH)E_2_: 2-hydroxy-estradiol; 2-MeOE_1_: 2-methoxy-estrone; 2-MeOE_2_: 2-methoxy-estradiol; 4-(OH)E_1_: 4-hydroxy-estrone; 4-(OH)E_2_: 4-hydroxy-estradiol; E_1_: estrone; E_1_S: estrone sulfate; E_2_: estradiol; genotoxic:total: [16α-hydroxy-estrone + 4-hydroxy-estradiol + 4-hydroxy-estrone]/total estrogens; 2-(OH)E_1_:16α-(OH)E_1_: 2-hydroxy-estrone to 16α-hydroxy-esterone ratio; 2E_1_-total:16α-total: [2-hydroxy-estrone + 2-methoxy-estrone] to [16α-hydroxy-esterone + estriol + 17-epiestriol] ratio; 2E_1_-total:4E_1_-total: [2-hydroxy-estrone + 2-methoxy-estrone] to [4-hydroxy-estrone + 4-methoxy-estrone] ratio; 2E_2_-total:4E_2_-total: [2-hydroxy-estradiol + 2-methoxy-estradiol] to 4-hydroxy-estradiol ratio; 2-total:4-total: [2-hydroxy-estrone + 2-hydroxy-estradiol + 2-methoxy-estrone + 2-methoxy-estradiol] to [4-hydroxy-estrone + 4-hydroxy-estradiol + 4-methoxy estrone] ratio. BW: body weight; C: control; CR: crossover; d: day(s); EX: exclusion criteria; FSH: follicle-stimulating hormone; HRT: hormone replacement therapy; I: intervention; IGF-1: insulin-like growth factor 1; IL-6: interleukin 6; IN: inclusion criteria; LH: luteinizing hormone; m: months; n.d.: no data available; *O*-DMA: *O*-desmethylangolensin; P: progesterone; RDA: recommended dietary allowances; SHBG: sex hormone binding globulin; TNF-α: tumor necrosis factor α; wk: weeks; y: years. ^1^ Participants who finished the study and for whom results were available; ^2^ according to personnel communication; ^3^ unclear which isoflavones were measured.

**Table 3:** Effects of isoflavones intake in women with different menopausal status on parameters related to the risk of breast cancer.

Study, Ref., Country	Participants	*n* ^1^	Study Design	Intervention/d; Isoflavone Intake/d	Duration (I)	Parameter	Results
Atkinson et al., 2004, [55], UK	IN: pre-, peri-, postmenopausal women, Wolfe P2 and DY mammographic breast patterns EX: OC, HRT, history of breast cancer, breast surgery	177	Parallel group, double-blind, placebo-controlled	Form: tablets I: 43.5 mg Isoflavones (1 mg genistein, 0.5 mg daidzein, 16 mg formononentin, 26 mg biochanin A) C: Placebo	12 m	Isoflavones (Σ genistein, daidzein, formononetin, biochantin A) (24-h-Urine: 0, 6, 12 m)	I > C (6 m, 12 m)
						FSH, LH, E_2_ (Serum: 0, 12 m)	ΔI = ΔC; baseline level not affected by genotype
						Gene polymorphisms of CYP17, CYP19, ESR1 (Lymphocytes: 0 m)	I = C
						Tyrosine kinase activity (Lymphocytes: 0, 12 m)	ΔI = ΔC
						Breast density (%) (Mammography: 0, 12 m)	ΔI = ΔC
Campbell et al., 2004, [56], UK	IN: pre- and postmenopausal women, aged 25–65 y EX: Premenopausal: OC, irregular MC, antibiotics ≤ 4 m; Postmenopausal: HRT, antibiotics ≤ 4 m, post oophorectomy, no amenorrhea ≥ 12 m	23	CR, double-blind, placebo-controlled, 2 m washout	Form: tablets I: 86 mg Isoflavones (8 mg genistein, 10 mg daidzein, 16 mg formononentin, 50 mg biochanin) C: Placebo	1 MC	Genistein, daidzein, equol Equol producer (24-Urine: 0, d28)	I > C 23% (pre), 20% (post)
						IGF-1, IGFBP-1, IGFBP-3 (Serum, premenopausal: 0, 1–3 d, 6–8 d 12–15 d, 21–23 d, 26–28 d; Serum, postmenopausal: 0, d28)	ΔI = ΔC (pre, post)
Powles et al., 2008, [58], UK	IN: pre-, peri-, postmenopausal women, first-degree relative with breast cancer EX: Pregnancy, breastfeeding, OC, HRT, history of breast cancer, other malignancy except basal cell carcinoma/cervical cancer in situ	401	Parallel group, double-blind, placebo-controlled, multicenter trial	Form: tablets I: 40 mg Isoflavones (genistein, daidzein, formonentin, biochanin; amounts unknown) C: Placebo	36 m	FSH (Blood: 0, 6, 12, 18, 24, 30, 36 m)	ΔI = ΔC
						Breast density (%) (Mammography: 0, 12, 24, 36 m)	ΔI = ΔC (all, pre, post)
Khan et al., 2012, [57], USA	IN: pre- and postmenopausal women, increased risk of breast cancer, history of unilateral minimal breast cancer risk; ≥4000 breast epithelial cells from rFNA EX: Pregnancy, breastfeeding, OC, HRT, soy foods during trial	98	Parallel group, double-blind, placebo-controlled	Form: capsules I: 235 mg Isoflavones (150 mg genistein, 74 mg daidzein, 11 mg glycitein) C: Placebo	6 m	Genistein, equol (Plasma: d11 of MC1 and MC3)	ΔI > ΔC (all, pre, post)
						Genistein, daidzein, equol (NAF: 0, 6 m)	I > C (genistein); results on daidzein and equol not mentioned, genistein in NAF and plasma not correlated
						E_2_, SHBG, E_2_/SHBG, FSH, P (Plasma: 0, 6 m)	ΔI = ΔC (all, pre, post)
						NAF volume, E_2_, cathepsin-D, EGF, IGF-1 (NAF: 0, 6 m)	ΔI = ΔC
						Gene expression of: BAX, BCL2, BCL3, BIRC5, CCND1, CDKN1A, CDKN2A, DDIT3, PTGS2, FAS, GBREB1, NFKB1, PARP-1, TP53 (genistein molecular targets), ESR1, ESR2, FOXA1, IGF1, IGFBP5, MYB, PGR, SCUBE, TFF1 (estrogen responsive genes), AR, PRLR, FGFR3, NDRG2, WNT5B (breast epithelial atypia associated genes) GAPDH, HPRT1 (housekeeping genes) (Mammary epithelial cells/rFNA; 0, 6 m)	Expression of all genes: ΔI = ΔC (all, pre, post)
						Ki-67 labeling index, atypical cells, Masood Score (Mammary epithelial cells/rFNA: 0, 6 m)	ΔI = ΔC (all, pre, post); Correlation Ki-67 labeling index and atypical cells

ΔI = ΔC: changes not significantly different between intervention and control treatment (no treatment effect). C: control; CR: crossover; d: day; E_2_: estradiol; EGF: epidermal growth factor; FSH: follicle-stimulating hormone; HRT: hormone replacement therapy; I: intervention; IGF-1: insulin-like growth factor 1; IGFBP-1: insulin-like growth factor binding protein 1; IGFBP-3: insulin-like growth factor binding protein 3; LH: luteinizing hormone; m: month(s); MC: menstrual cycle; NAF: nipple aspirate fluid; OC: oral contraceptive; post: postmenopausal; pre: premenopausal; P: progesterone; rFNA: random fine needle aspiration; SHBG: sex hormone binding globulin. ^1^ Participants who finished the study and for whom results were available; otherwise the number of participants who were randomized.

**Table 4:** Criteria to assess the risk of bias in studies with premenopausal women.

	Nagata et al., 1998 [32]	Duncan et al., 1999 [31]	Brown et al., 2002 [30]	Kumar et al., 2002 [33]	Maskarinec et al., 2002a [34]	Maskarinec et al., 2002b [35]	Maskarinec et al., 2003 [37]	Maskarinec et al., 2004a [36]	Maskarinec et al., 2004b [38]	Zittermann et al., 2004 [39]	Maskarinec et al., 2005 [40]	Maskarinec et al., 2009b [41]	Maskarinec et al., 2011a [42]	Maskarinec et al., 2011b [43]	Maskarinec et al., 2012, S1 [44]	Maskarinec et al., 2012, S2 [44]	Morimoto et al., 2012 [45]	Sen et al., 2012 [46]	Maskarinec et al., 2013 [47]	Maskarinec et al., 2017, S1 [48]	Maskarinec et al., 2017, S2 [48]
Randomization																					
List generated ^1^	√	√	√	√	√	√	√	√	√	√	√	√	√	√	√	√	√	√	√	√	√
Adequate randomization method	?	?	?	√	?	?	?	?	?	?	?	?	?	?	?	?	?	?	?	?	?
Allocation concealment	?	?	?	?	?	?	?	?	?	?	?	?	?	?	?	?	?	?	?	?	?
Blinding																					
Participants	×	?	√	√	√	√	√	×	×	?	×	×	×	×	×	×	×	×	×	×	×
Investigators	?	?	?	√	√	√	√	√	?	?	?	?	?	?	?	?	?	?	?	?	?
Outcome assessments	√	√	√	√	√	√	√	√	√	√	√	√	√	√	√	√	√	√	√	√	√
Considering potential confounders																					
Nutritional behavior	√	√	√	√	√	√	√	√	√	√	√	√	√	√	√	√	√	√	√	√	√
Dietary restrictions	√	√	×	√	?	?	?	√	√	×	√	√	√	√	√	√	√	√	√	√	√
Compliance assessed	√	×	√	√	√	√	√	√	√	√	√	√	√	√	√	√	√	√	√	√	√
Study protocol																					
Registered	?	?	?	?	?	?	?	?	?	?	?	?	√	√	?	√	√	√	√	?	√
Outcomes reported ^2^	?	?	?	?	?	?	?	?	?	?	?	?	√	√	?	√	√	√	√	√	?
Results																					
Dropouts/missing data	√	?	√	√	√	?	?	√	√	√	√	√	√	√	√	√	√	√	√	√	√
Dropouts reported ^3^	√	−	×	√	×	?	?	√	√	√	√	√	√	√	√	×	×	×	√	√	√
Reasons for dropouts/missing data ^4^	×	−	×	×	×	?	?	×	×	×	×	×	×	×	×	×	×	×	×	×	×
Intention-to-treat analysis ^5^	×	−	×	√	?	?	?	?	?	?	?	?	?	?	?	?	?	?	?	√	√

×: No; not considered; √: yes, considered; ?: not clear, no details available, −: irrelevant; S: study. ^1^ Before the start of the study; ^2^ reported according to registration; ^3^ reported separately for each group or treatment and being comparable between groups, ^4^ reported and being comparable between groups or treatments, ^5^ missing data were imputed by appropriate statistical models.

**Table 5:** Criteria to assess the risk of bias in studies with postmenopausal women.

	Xu et al., 2000 [50]	Verheus et al., 2008 [49]	Maskarinec et al., 2009a [51]	Steinberg et al., 2011 [52]	Delmanto et al., 2013 [53]	Nadadur et al., 2016 [54]
Randomization						
List generated before start of the study	√	√	√	√	√	√
Adequate randomization method	?	?	√	√	√	?
Allocation concealment	?	?	?	√	√	?
Blinding						
Participants	?	√	√	√	√	√
Investigators	?	√	√	√	√	?
Outcome assessments	√	√	√	√	√	√
Considering potential confounders						
Nutritional behavior	√	×	√ ^1^	×	×	√
Dietary restrictions	√	×	?	×	×	√
Compliance assessed	√	√	√	√	√	√
Study protocol						
Registered	?	?	?	√	?	?
Outcomes reported according to registration	?	?	?	×	?	?
Results						
Dropouts/missing data	√	√	√	√	√	√ ^2^
Dropouts/missing data reported ^3^	√	√	√	√	×	√
Reasons for dropouts/missing data reported ^4^	×	×	×	√	×	√
Intention-to-treat analysis ^5^	×	√	√	√	×	−

×: No, not considered; √: yes, considered; ?: not clear, no details available, − irrelevant. ^1^ Investigated but results not given; ^2^ due to inclusion of subjects whose premenopausal status was not confirmed by hormone analysis; ^3^ separately for each group and dropout comparable between groups; ^4^ separately for each group or treatment and being comparable between groups; ^5^ missing data were imputed by appropriate statistical models.

**Table 6:** Criteria to assess the risk of bias in studies with a mixed group of pre-, peri- and postmenopausal women.

	Atkinson et al., 2004 [55]	Campbell et al., 2004 [56]	Powles et al., 2008 [58]	Khan et al., 2012 [57]
Randomization				
List generated before start of the study	√	√	√	√
Adequate randomization method	√	?	√	√
Allocation concealment	?	?	√	?
Blinding				
Participants	√	√	√	√
Investigators	√	√	√	√
Outcome assessments	√	√	√	√
Considering potential confounders				
Nutritional behavior	×	√	×	√
Dietary restrictions	×	√	×	×
Compliance assessed	√	√	×	√
Registration of the study protocol				
Registered	?	?	?	√
Outcomes reported according to registration	?	?	?	√
Results				
Dropouts/missing data	√	×	√	√
Dropouts/missing data reported ^1^	√	−	×	×
Reasons for dropouts/missing data reported ^2^	×	−	×	?
Intention-to-treat analysis ^3^	×	−	×	×

×: No, not considered; √ yes, considered; ? not clear, no details available. ^1^ Separately for each group or treatment and being comparable between the groups or treatments; ^2^ separately for each group or treatment and being comparable between the groups or treatments; ^3^ missing data were imputed by appropriate statistical models.
